# Subthalamic nucleus deep brain stimulation for Parkinson’s disease: 8 years of follow-up

**DOI:** 10.1186/2047-9158-2-11

**Published:** 2013-05-24

**Authors:** Dianyou Li, Chunyan Cao, Jing Zhang, Shikun Zhan, Shengdi Chen, Bomin Sun

**Affiliations:** 1 Department of Functional Neurosurgery, Ruijin Hospital, Shanghai Jiaotong University School of Medicine, Shanghai 200025, China; 2 Department of Neurology, Ruijin Hospital, Shanghai Jiaotong University School of Medicine, Shanghai 200025, China

**Keywords:** Deep brain stimulation, Long-term effects, Parkinson’s disease, Subthalamic nucleus

## Abstract

**Objective:**

The short-term benefits of bilateral stimulation of the subthalamic nucleus (STN) in patients with advanced Parkinson’s disease (PD) are well documented, but long-term benefits are still uncertain. The aim of this study is to evaluate the outcome of 8 years of bilateral STN stimulation to PD patients.

**Methods:**

In this study, 31 consecutive PD patients were treated with bilateral STN stimulation. Their functional status was measured using the Activities of Daily Living section of the Unified Parkinson’s Disease Rating Scale (UPDRS-ADL) at drug on (with medication) and drug off (without medication) states preoperatively and at 1, 5, and 8 years postoperatively. In addition, Levodopa equivalent doses and stimulation parameters were also assessed.

**Results:**

After 8 years of STN stimulation, the UPDRS-ADL scores were improved by 4% at drug off status (*P* > 0.05) and 22% at drug on status (*P* < 0.05) compared with baseline; the levodopa daily doses were reduced by 28% (*P* < 0.05) compared with baseline; the stimulation voltage and pulse width were not changed, but the stimulation frequency was decreased remarkably compared with the 5 years of follow-up. Adverse events were observed in 6 patients, including misplacement of the electrode and skin erosion requiring further surgery. All events were resolved without permanent sequelae. 2 patients died of aspiration pneumonia 6 and 7 years after surgery.

**Conclusions:**

The marked improvement in UPDRS-ADL scores were still observed after 8 years of bilateral STN stimulation with medication.

## Introduction

Chronic high-frequency deep brain stimulation (DBS) has been a standard surgical treatment to the Parkinson’s disease (PD) patients. The subthalamic nucleus (STN) stimulation can both alleviate the parkinsonian motor disability and reduce the daily dose of levodopa [1,2]. So far, it has not been possible to definitively determine whether the effectiveness of DBS decays over time or if DBS generates neuroprotective effect [3], since only limited recordings on the long-term clinical course of the patients with DBS surgery are available [4,5].

Between 1999 and 2011, 304 levodopa responsive PD patients were treated with STN stimulation in Ruijin Hospital, Shanghai Jiaotong University School of Medicine. Compared with preoperative drug off condition, the activities of daily living scores (Unified Parkinson Disease Rating Scale II (UPDRS-ADL)) of 93% patients showed a 50% improvement after 1 year of STN stimulation, which were evaluated after an overnight withdrawl of levodopa (stimulation on and drug off condition). Additionally, the average levodopa equivalent doses were reduced by 45% after 1 year of STN stimulation (not published). Herein, we present the results of functional status, levodopa equivalent dosages and stimulation parameters obtained from a series of 31 consecutive PD patients with STN stimulation before surgery and at 1, 5 and 8 years after surgery.

## Materials and methods

### Patients

During the period of January 2000 and August 2003, 31 consecutive PD patients (22 men and 9 women, mean age 53.5 ± 11.7 years) were operated with bilateral STN stimulation. The selection criteria were: (i) clinically diagnosed idiopathic PD patients with Hoehn and Yahr scale (stage 2 to stage 4) [6,7]; (ii) >35% improvement in motor symptoms in response to levodopa treatment [8]. The study was carried out after the local institutional review board approval was obtained and all participants provided their written informed consent. None of the patients was demented and depressed as determined by neuropsychological assessment. 2 patients died of aspiration pneumonia 6 and 7 years after surgery.

### Surgical procedure

The neurosurgical procedure was performed as previously described [9]. The intended target coordinates were determined on the basis of 1.5 T magnetic resonance imaging(MRI), then the electrodes (model 3387–40, 7428, Medtronic, Minneapolis, MN, USA) were stereotactially implanted into the bilateral STN under local anesthesia. Intraoperative macroelectrode stimulation was used in confirming the target position. At last the electrodes were connected to the extension wires (7482, Medtronic) and the programmable pulse generator (IPG) (bilateral Itrel® II, unilateral Kinetra; Medtronic) was implanted subclavicularly under general anesthesia. IPG programming was initiated on the following day. Electrical parameters (voltage, pulse width, and frequency) were progressively adjusted using an electromagnetic programmer (7532, 8840 neurological programmer; Medtronic).

### Clinical evaluation

The quality of life was assessed with UPDRS-ADL scores preoperatively (baseline) and at 1, 5 and 8 years postoperatively at drug off state after overnight withdrawl of antiparkinsonian medication and drug on state after the administration of a single suprathreshold dose of levodopa (150% of the usual effective dose taken in the morning), respectively. The percentage improvements of UPDRS-ADL scores at 1, 5 and 8 years after surgery were calculated compared with baseline at drug off and drug on statuses, respectively. The levodopa equivalent dosages and stimulation parameters were also documented. The UPDRS-ADL assessments were scored through clinic interview and telephone interview by a blinded rater (DL). Anti-parkinsonnian drugs and doses were recorded at each interview and transformed into levodopa equivalent daily doses [5]. All the evaluations after surgery were performed when PD patients were in routine stimulation.

### Statistical analysis

Data were expressed as mean ± SD. Statistical analysis was performed by repeated measures analysis of variance followed by Bonferroni. SPSS (version13.0, SPSS, Chicago, IL, USA) was used for the statistical analysis. The significance levels were set at P < 0.05.

## Results

### Effects of continuous bilateral STN stimulation on the quality of life of PD patients

Compared with the drug off baseline, the ADL scores were improved by 58%, 33%, and 4% at 1, 5 and 8 years postoperatively under stimulation on and drug off status. Compared with the drug on baseline, the ADL scores were improved by 69%, 49%, and 22% at 1, 5 and 8 years postoperatively under stimulation on and drug on status (Table 1). These findings indicate that with STN stimulation, the quality of life of patients was better than baseline at 1 and 5 years under drug off condition, and the effect didn’t remain 8 years. However, the marked improvement of UPDRS-ADL scores still remained at 8 years postoperatively under stimulation on and drug on condition.

**Table 1:** **Activities of daily living scores and levodopa equivalent doses before surgery** (**baseline**) **and 1 year**, **5 years and 8 years after surgery and the percentage change compared with baseline**

		**Baseline**	**1 year**	**5 years**	**8 years**
UPDRS-ADL scores	Drug off	21.5 ± 8.1	9.2 ± 4.3 (58%)*	14.4 ± 6.0 (33%)*#Ω	20.1 ± 8.6 (4%)#ΩΨ
(Improvement percentage)	Drug on	8.8 ± 4.3*	6.4 ± 3.0 (69%)*	10.9 ± 5.5 (49%)*ΔΩ	16.7 ± 7.6(22%)*Δ#ΩΨ
LED (Increase percentage)	mg/day	967.8 ± 381.3	457.2 ± 283.7 (53%)#	573.0 ± 383.0 (40%)#	669.1 ± 480.69 (28%)#Ω

Values are means (SD).

* p significant compared with drug off condition before surgery.

# p significant compared with drug on condition before surgery.

Δ p significant compared with drug off condition at the same time of follow-up.

Ω p significant compared with corresponding assessment 1 year after surgery.

Ψ p significant compared with corresponding assessment 5 years after surgery.

*LED*: Levodopa equivalent doses.

The UPDRS-ADL scores appeared more improvement under STN stimulation and drug treatment than sole stimulation at 5 and 8 years after surgery, but not at 1 year after surgery.

### Medications and stimulation settings

Compared with preoperative condition, the daily dose of levodopa equivalent was reduced by 53%, 40%, and 28% at 1, 5 and 8 years after surgery, respectively (Table 1).

Monopolar stimulation with the use of single or double contacts from the quadripolar electrode was applied in 90% of the patients. Compared with 1 month after surgery, there are remarkable increases of the mean voltage and pulse width of stimulation at 1, 5 and 8 years, but the frequency of stimulation showed obvious decrease at 5 and 8 years. Compared with 1 year after surgery, the mean voltage of stimulation was increased, but the frequency of stimulation was decreased and the pulse width of stimulation was not changed at 5 and 8 years. Relative to 5 years after surgery, there were no change in the mean voltage and pulse width of stimulation, but there was significant decrease in the frequency of stimulation at 8 years (Table 2).

**Table 2:** **Stimulation parameters at 1 month**, **1 year**, **5 years and 8 years of follow**-**up**

**Stimulation parameters**	**1 month**	**1 year**	**5 years**	**8 years**
Amplitude (V)	2.2 ± 0.3 (1.6-2.7)	2.6 ± 0.4 (1.9-3.3) ※	2.7 ± 0.4 (1.9-3.5)※#	2.8 ± 0.4 (2.2-3.6)※#
Pulse width (μs)	60.0 ± 0.0 (60–60)	75.8 ± 15.2 (60–90)※	80.8 ± 17.3 (60–120)※	80.8 ± 17.3 (60–120)※
Rate (Hz)	163.2 ± 14.2 (130-185)	159.9 ± 13.3 (130–185)	150.1 ± 12.2 (130–160)※#	141.3 ± 14.6 (85-160)※#Δ

Data presented as mean ± SD (range).

※ p significant compared with 1 month after surgery.

# p significant compared with 1 year after surgery.

△ p significant compared with 5 years after surgery.

### Adverse events

2 patients died of aspiration pneumonia due to swallow disorders 6 and 7 years after surgery. In 4 patients, malposition of the electrodes was revealed by ineffectiveness of stimulation and MRI, and the electrodes were adjusted to alleviate the symptoms. 2 patients had the skin erosion in the IPG pocket and the stimulators were repositioned. There was no symptomatic intracerebral hemorrhage happened in this study.

## Discussion

In this study, we provide 8 years of follow-up of PD patients with bilateral STN stimulation. Our results indicate that the improvement of functional status in PD patients by sole STN stimulation was sustained 5 years after surgery; and this effect declined at 8 years after surgery. However, compared with preoperative baseline status, the quality of life was still improved remarkably after 8 years of stimulation and antiparkinsonian medication, furthermore, the levodopa equivalent dosage is still lower than that used at preoperative status.

As previously reported, axial signs like postural instability and gait disorders of the patients showed the most striking progressive loss of benefits from stimulation over time [5,10]. These symptoms have great impact on the quality of life of PD patients, so the decline in DBS benefit at the 8-year follow-up might be due to the disease progression of PD [11] and thus suggests that the STN stimulation is lack of remarkable hindrance to the progression of PD [10].

The daily doses of levodopa were reduced markedly and the stimulation voltage and pulse width were increased to improve the patients symptoms within 5 years of stimulation. 5 years later, the aggravation of symptoms can’t be compensated by the adjustment of the stimulation parameters due to the intolerable side effects caused by high intensity stimulation. The improvement by medication was still observed since stimulation can ameliorate some motor complications of medications [12], unlike the observation of other groups that PD patients showed a remarkable progressive loss of levodopa responsiveness over the years [13,14]. In our study, the patients of younger-onset (53.5 years) and relatively shorter duration (7.86 years) might contain more dopaminergic reactive receptors in striatum, which bring about the consistent responsiveness to levodopa over the years, and also resulted in less surgical complications relative other study [15].

Compared with 5 years of follow-up, stimulation voltage and pulse width were not changed at the 8 years of follow-up, but the stimulation frequency was reduced. At the beginning of stimulation, high frequency (160-185 Hz) was applied to control the tremor, one of the most prominent symptoms of PD; after the tremor was finally restrained with long-term of stimulation [16], the relative lower frequency (130-145 Hz) was applied. Additionally, when some of the patients developed the axial symptoms like swallow, speech and gait disorders, the stimulation frequency was reduced (85-115 Hz) further to improve the balance and speech functions, especially in some bradykinesia or rigidity dominated patients, which is in consistent to the recently reported studies [17,18].

Our study has several limitations, including the lack of a PD group with sole medication therapy and double-blinded assessments, and the lack of stimulation off assessment since most of PD patients can’t endure the stimulation wash-out period which means stimulation is turned off at least 2 hours [12]. Because data of UPDRS III motor scores of 7 patients at 5 years of follow-up have not been reached until now, the UPDRS III motor scores are not reported in this study.

## Conclusion

Our findings confirm the continuous improvement in the functional status of PD patients by 8 years of bilateral STN stimulation, combined with antiparkinsonian medication. In our study, the marked efficacy of STN stimulation in meliorating the functional status of parkinsonian disability results primarily from the strict selection of levodopa responsive patients and the careful management of the medication and IPG programming after surgery by a multidisciplinary team.

## Abbreviations

DBS: Chronic high-frequency deep brain stimulation; STN: The subthalamic nucleus; PD: Parkinson’s disease; ADL: An activity of daily living; UPDRS: Unified Parkinson Disease Rating Scale; MRI: Magnetic resonance imaging.

## Competing interests

The authors declare that there is no conflict of interest.

## Authors’ contributions

BS and SC contributed to the design of the study and revision of the manuscript; DL acquired the data and wrote the first draft of the article; CC designed and performed the statistical analysis, and programmed IPG; BS and SZ operated the surgery; JZ managed the patients. All of the authors have read and approved the final manuscript.

## References

[B1] Limousin P Krack P Pollak P Benazzouz A Ardouin C Hoffmann D Benabid AL Electrical stimulation of the subthalamic nucleus in advanced Parkinson's disease N Engl J Med 1998 339 1105 1111 10.1056/NEJM199810153391603 9770557

[B2] Volkmann J Allert N Voges J Weiss PH Freund HJ Sturm V Safety and efficacy of pallidal or subthalamic nucleus stimulation in advanced PD Neurology 2001 56 548 551 10.1212/WNL.56.4.548 11222806

[B3] Carvalho GA Nikkhah G Subthalamic nucleus lesions are neuroprotective against terminal 6-OHDA-induced striatal lesions and restore postural balancing reactions Exp Neurol 2001 171 405 417 10.1006/exnr.2001.7742 11573992

[B4] Lyons KE Pahwa R Long-term benefits in quality of life provided by bilateral subthalamic stimulation in patients with Parkinson disease J Neurosurg 2005 103 252 255 10.3171/jns.2005.103.2.0252 16175854

[B5] Krack P Batir A Van Blercom N Chabardes S Fraix V Ardouin C Koudsie A Limousin PD Benazzouz A LeBas JF Five-year follow-up of bilateral stimulation of the subthalamic nucleus in advanced Parkinson's disease N Engl J Med 2003 349 1925 1934 10.1056/NEJMoa035275 14614167

[B6] Defer GL Widner H Marie RM Remy P Levivier M Core assessment program for surgical interventional therapies in Parkinson's disease (CAPSIT-PD) Mov Disord 1999 14 572 584 10.1002/1531-8257(199907)14:4<572::AID-MDS1005>3.0.CO;2-C 10435493

[B7] Hoehn MM Yahr MD Parkinsonism: onset, progression and mortality Neurology 1967 17 427 442 10.1212/WNL.17.5.427 6067254

[B8] Deep-brain stimulation of the subthalamic nucleus or the pars interna of the globus pallidus in Parkinson's disease Deep-brain stimulation of the subthalamic nucleus or the pars interna of the globus pallidus in Parkinson's disease N Engl J Med 2001 345 956 963 11575287 10.1056/NEJMoa000827

[B9] Bomin S kangyong Liu LL Li P Dianyou L Zhipei L Daokuan L Bilateral deep brain stimulation of the subthalamic nucleus in advanced Parkinson’s disease Chin J Neurosurg 2002 18 8 11

[B10] Fasano A Romito LM Daniele A Piano C Zinno M Bentivoglio AR Albanese A Motor and cognitive outcome in patients with Parkinson's disease 8 years after subthalamic implants Brain 2010 133 2664 2676 10.1093/brain/awq221 20802207

[B11] Moro E Lozano AM Pollak P Agid Y Rehncrona S Volkmann J Kulisevsky J Obeso JA Albanese A Hariz MI Long-term results of a multicenter study on subthalamic and pallidal stimulation in Parkinson's disease Mov Disord 2010 25 578 586 10.1002/mds.22735 20213817

[B12] Temperli P Ghika J Villemure JG Burkhard PR Bogousslavsky J Vingerhoets FJ How do parkinsonian signs return after discontinuation of subthalamic DBS? Neurology 2003 60 78 81 10.1212/WNL.60.1.78 12525722

[B13] Piboolnurak P Lang AE Lozano AM Miyasaki JM Saint-Cyr JA Poon YY Hutchison WD Dostrovsky JO Moro E Levodopa response in long-term bilateral subthalamic stimulation for Parkinson's disease Mov Disord 2007 22 990 997 10.1002/mds.21482 17443692

[B14] Moro E Esselink RJ Benabid AL Pollak P Response to levodopa in parkinsonian patients with bilateral subthalamic nucleus stimulation Brain 2002 125 2408 2417 10.1093/brain/awf249 12390968

[B15] Castrioto A Lozano AM Poon YY Lang AE Fallis M Moro E Ten-year outcome of subthalamic stimulation in Parkinson disease: a blinded evaluation Arch Neurol 2011 68 1550 1556 10.1001/archneurol.2011.182 21825213

[B16] Goetz CG Stebbins GT Blasucci LM Differential progression of motor impairment in levodopa-treated Parkinson's disease Mov Disord 2000 15 479 484 10.1002/1531-8257(200005)15:3<479::AID-MDS1009>3.0.CO;2-P 10830412

[B17] Moreau C Defebvre L Destee A Bleuse S Clement F Blatt JL Krystkowiak P Devos D STN-DBS frequency effects on freezing of gait in advanced Parkinson disease Neurology 2008 71 80 84 10.1212/01.wnl.0000303972.16279.46 18420482

[B18] Brozova H Barnaure I Alterman RL Tagliati M STN-DBS frequency effects on freezing of gait in advanced Parkinson disease Neurology 2009 72 770 771 10.1212/01.wnl.0000339385.187472.7d 19237711

